# Performance of Ag-doped CuO nanoparticles for photocatalytic activity applications: Synthesis, characterization, and antimicrobial activity

**DOI:** 10.1186/s11671-024-04108-3

**Published:** 2024-10-05

**Authors:** Ahmed T. Mosleh, Elbadawy A. Kamoun, Shahira H. EL-Moslamy, Samar A. Salim, Heba Y. Zahran, Samer H. Zyoud, Ibrahim S. Yahia

**Affiliations:** 1 Nanotechnology Section, Egyptian Company for Carbon Materials, El-Sheraton/El-Nozha, 11757 Cairo Egypt; 2 Department of Chemistry, College of Science, King Faisal University, 31982 Al-Ahsa, Saudi Arabia; 3 Polymeric Materials Research Department, Advanced Technology and New Materials Research Institute (ATNMRI), City of Scientific Research and Technological Applications (SRTA-City), New Borg Al-Arab City, 21934 Alexandria Egypt; 4 Bioprocess Development Department (BID), Genetic Engineering and Biotechnology Research Institute (GEBRI), City of Scientific Research and Technological Applications (SRTA-City), New Borg El-Arab City, 21934 Alexandria Egypt; 5 Nanotechnology Research Center (NTRC), The British University in Egypt (BUE), El-Sherouk City, 11837 Cairo Egypt; 6 Central Labs, King Khalid University, PO Box 960, AlQura’a, Abha, Saudi Arabia; 7 Laboratory of Nano-Smart Materials for Science and Technology (LNSMST), Department of Physics, Faculty of Science, King Khalid University, PO Box 9004, 61413 Abha, Saudi Arabia; 8 Department of Mathematics and Sciences, Ajman University, Ajman, United Arab Emirates; 9 Center of Medical and Bio-Allied Health Sciences Research (CMBHSR), Ajman University, P.O. Box 346, Ajman, United Arab Emirates

**Keywords:** Ag/CuO nanoparticles, Antimicrobial activity, Photocatalytic activity, Photodegradation

## Abstract

The auto-combustion method synthesized CuO NPs and Ag/CuO NPs. The Ag/CuO NPs were analyzed using Fourier-transform infrared, X-ray diffraction, scanning electron microscope, and Energy-dispersive X-ray spectroscopy instrumental analyses. The energy band gap, as determined by DRS properties, decreases from 3.82 to 3.50 eV for pure CuO and 10% Ag/CuO NPs, respectively. The photodegradation efficiency of Rhodamine-B & Carmine by 10% Ag/CuO NPs was nearly 98.9 and 97.8%, respectively. Antimicrobial trials revealed that the antimicrobial efficacy of Ag/CuO NPs at several dosages (20, 40, 60, 80, 100, and 120 µg/mL) against human pathogens was initially assessed using the agar well-diffusion method, and then the broth dilution method. Noticeably, the minimum inhibitory concentration of Ag/CuO NPs for all pathogens ranged from 100 to 120 µg/ml, was determined. Generally, the observed minimum microbicide concentration has a wide range of Ag/CuO NPs doses, ranging from 150 to 300 µg/ml, which helps kill (99.99%) all tested pathogenic cells. The largest relative inhibitory activities (%) were recorded against *Escherichia coli* (81.45 ± 1.39) at 120 g/mL of Ag/CuO NPs and 100 μg/mL (80.43 ± 0.59), followed by 80 µg/mL (72.33 ± 0.82). Additionally, the lowest relative inhibitory activities (%) were monitored versus fungal cells and Gram-positive bacteria at 120 µg/mL of Ag/CuO NPs as 52.17 ± 1.49 and 53.42 ± 1.71; respectively.

**Graphic abstract:**

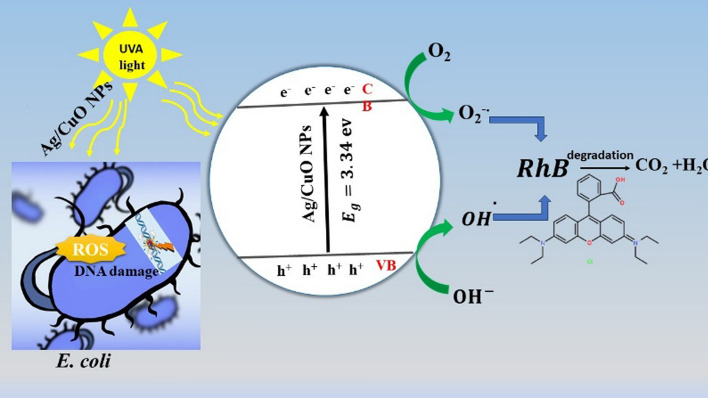

## Introduction

Recently, environmental protection against pollution has encountered a huge challenge that concerns humanity. Pollutant removal from water by semi-conducted materials has attracted much interest from environmentalists [1–3]. However, the textile, printing, and dyeing sectors use a significant amount of water, and their yearly wastewater discharge makes up over 70% of the global discharge. Azo dyes seriously threaten the environment, aquatic life, and human life because of their intense chromaticity, toxicity, and stable azo functional groups (N=N) and aromatic rings. These dyes are very difficult to biodegrade [4]. Since pollution rates exceed natural water reservoirs' capacity for self-purification, water pollution is a great concern. About Every year, 0.7 million tons of dyes are produced worldwide, with 10–15% of those dyes ending up in aquatic bodies [5].

Therefore, dye removal from wastewater is needed to lessen this environmental concern. Several methods have been tried to identify suitable solutions, such as biological treatment [6], precipitation [7], electrochemical techniques [8], and photocatalysis [9]. One of the most modernist techniques, catalytic photo-degradation, is a potential method as it can be operated without expensive oxidants at low pressure and temperature by stable and low-cost catalysts [10–12]. The photo-catalysts absorb solar energy that is identical to or more than the bandgap of the photo-catalyst materials, leading to the excitation of the pairs of electron holes (e–h) in the process of photocatalysis for degrading organic dyes in water. These pairs transfer to the photocatalysts' surface and then initiate a sequence of reactions with the surrounding medium, producing reactive species responsible for removing contaminants [13].

Organic dyes are widely utilized in numerous industrial applications, such as coating, photochemical industries, photography, and textile dyeing. Many dyes consumed as painting agents harm marine creatures [14]. Rhodamine-B (RhB) is a commonly used xanthene dye in textile industries [15]. The existing water treatment process cannot eliminate it due to its complex structure [16]. It is more stable photothermally, making it harder to break down and, as a result, harder to control the environmental hazard it poses. RhB is thought to cause animal toxins that are carcinogenic, genotoxic, and neurotoxic [17]. Reductive amination of RhB produces carcinogenic amines due to anaerobic degradation [18]. The solubility of anionic dyes, such as the inexpensive red pigment carmine (carminic acid) derived from cochineal and aluminum. It belongs to the Coccidia family [19]. Carmine dye, also known as E 120, is widely used as a flavoring in cosmetics and is frequently utilized in the food sector. It has a specific place in the textile industry and is utilized in the pharmaceutical, plastic, and natural colorant industries [20].

RhB has been widely employed to evaluate the photocatalytic degradation process because it is a common organic dye in industrial wastes and can be selectively oxidized to construct Rh-110 [21–23]. As a typical azo dye, carmine is chemically stable and hard to biodegrade due to its benzene ring and highly conjugated molecular system[24, 25]. The degradation products of carmine dye are known to be highly poisonous, mutagenic, carcinogenic, and allergic, posing a hazard to aquatic life and causing various health problems [26]. Another environmental issue is the interaction between carmine dye and metal ions, which can change their physiochemical characteristics, stability, and toxicity levels [27].

Contained by the AOP process, heterogeneous photocatalysis offers cost-effective removal of pollutants, dyes, and organic compounds from wastewater, exhibiting stability and significant potential. Photocatalysis with metal-doped heterogeneous catalysts is the most effective method for removing oxidative processes from wastewater [28]. The oxidative process involving metal coupling exhibits low intensity, molecular mass, and environmental toxicity. This process enhances and modifies the energy of charge carriers, leading to high absorption. The formed reactive species result from the metal ions' oxidative process and catalytic reaction.

The prevention of harmful infectious diseases has grown significantly as a global concern because of bacterial resistance to drugs. Alternatively, abundant antibiotics and antifungal formulations are accessible to manage infections resulting from bacteria [29, 30]. Because of their unique chemical or physical properties and high surface area-to-volume ratio, nanoscale materials are currently being exploited as novel antibacterial agents [31]. So, the Ag-based nanocomposite is also very good at preventing microbial contamination in wastewater because it naturally has antibacterial properties. The antibacterial properties of silver nanoparticles, which stop the growth of certain pathogenic bacteria, are well known. Long-lasting antimicrobial effects and the prevention of biofilm formation are provided by incorporating Ag nanoparticles into the CuO matrix, which lowers the danger of microbial renewal and biofouling in wastewater treatment systems [32]. CuO NPs are inexpensive when contrasted with gold and silver nanoparticles used as antimicrobial agents [33].

In the category of metal as well as metal oxide nanoparticles, like copper oxide nanoparticles (CuO NPs), high conductivity, high binding energy, and high thermal conductivity values exist.

Among these, CuO's non-toxicity and ease of synthesis make it a great option for a semiconductor photocatalyst. In addition, it possesses excellent optical and electrical qualities, high thermal stability, conductivity, and exciton binding energy values [34]. The widespread utilization of CuO NPs in a heterogeneous photocatalytic-AOP method is attributed to their redox potential and electron transfer process, in addition to p-type material with a narrow band gap [35, 36]. The collaboration of CuO with precious metals is an exciting area of study due to their improved biomedical, electronic, photocatalytic, and other uses. For instance, in the scenario of CuO-adorned Ag nanoparticles, the charge transfer occurs from CuO to Ag NPs, inhibiting the recombination of charge carriers and consequently demonstrating efficient photocatalytic properties. [37–39]. CuO nanoparticles have been observed to enhance biological functions due to their rapid nucleation growth, reduced size, and interaction with catalytic and biological substances [40].

Our study describes an auto-combustion technique for creating CuO and Ag/CuO NPs. A range of analyses were employed to examine the chemical structure, crystalline nanostructures, surface morphologies, compositions, and optical features of the produced substances. Photocatalytic execution of the fabricated nanocomposites was estimated by the most extensively used RhB and carmine dye degradation underneath simulated UVA-light irradiation. In addition, Ag/CuO NPs can rapidly penetrate bacteria's nuclear material depending on their size, shape, and surface area, resulting in relatively high antimicrobial activity.

## Materials and methods

### Materials

A list of chemicals with specifications that were employed are displayed in Table 1.

**Table 1:** Materials information regarding the molecular structure, purity, and supplier for each chemical used

Materials	Molecular structure	Purity (%)	Supplier
Copper nitrate trihydrate	Cu (NO_3_)_2_.3H_2_O	99	LOBA Chem, India
Silver nitrate	AgNO_3_	99.98	Merck, Germany
Starch	C_6_H_10_O_5_)_n_	95	Sigma Aldrich, Germany
Sodium chloride	NaCl	98	LOBA Chem, India
Sodium nitrate	NaNO_3_	98	LOBA Chem, India
Ascorbic acid	C_6_H_8_O_6_	99.98	Sigma Aldrich, Germany
Isopropyl alcohol	C_3_H_8_O	99.5	Alfa Asear, India
Rhodamine B dye	C_28_H_31_ClN_2_O_3_	≥ 95	Merck, Germany
Carmine dye	C_22_H_20_O_13_	85	Sigma Aldrich, Germany

### Manufacture of Ag/CuO NPs

**Scheme 1:**
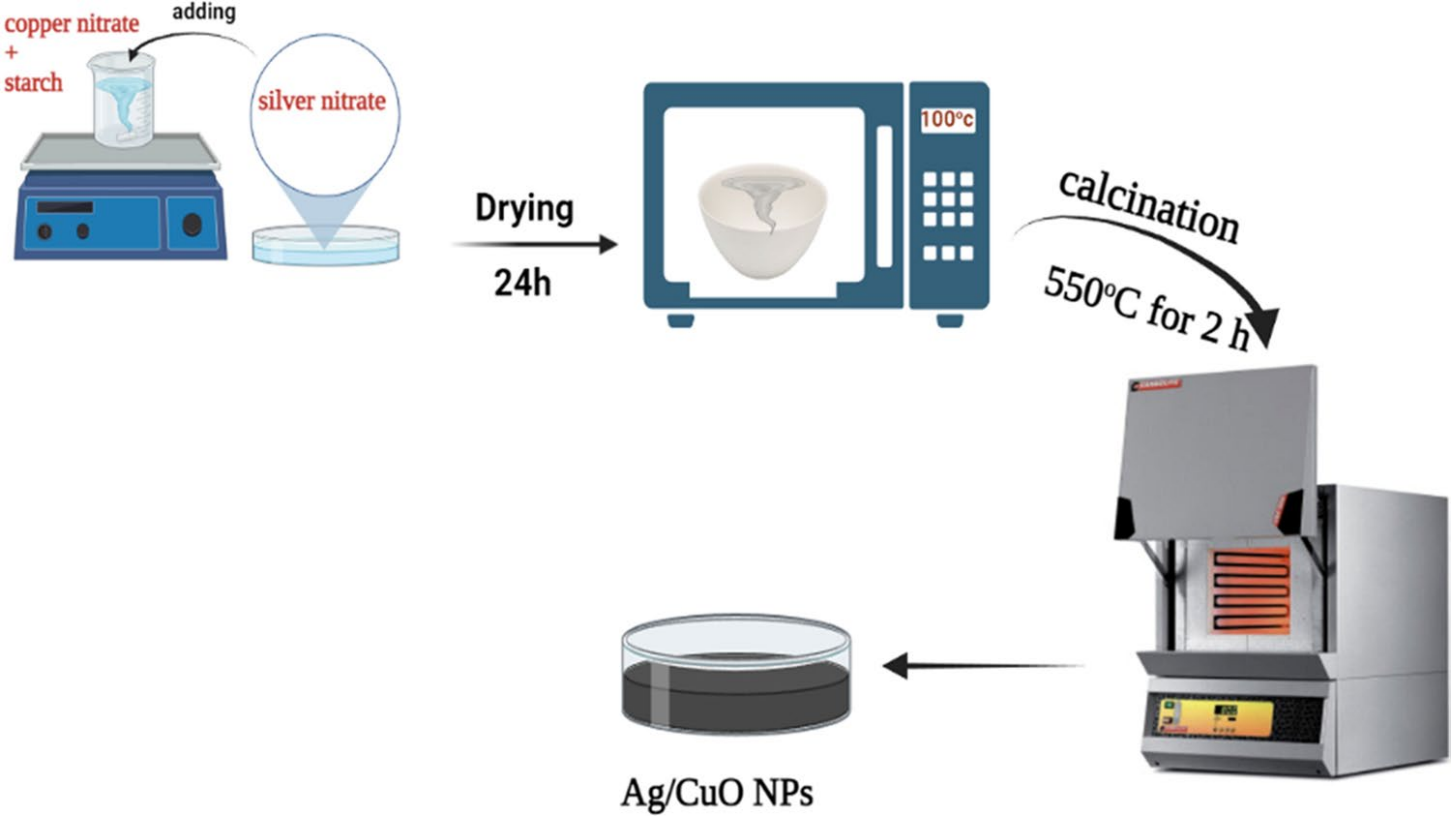
Representation diagram for the manufacture of Ag/CuO NPs

The Ag/CuO nanoparticles were produced using the auto-combustion method. Initially, 10g of copper nitrate and 10 g of starch were thoroughly mixed in 30 mL of distilled water. Subsequently, varying amounts of silver nitrate (0, 1, 3, 5, 10, and 30 wt.%) were added to 6 crucibles and blended well. The crucibles were then wiped in a stove for 24 h at 100 °C and calcined for nearly 2 h at 550 °C to obtain a catalyst, as depicted in Scheme 1.

The reaction mechanism for synthesis of Ag/CuO nanoparticles is as follows:

$$ {\text{Cu }}\left( {{\text{NO}}_{3} } \right)_{2} .{\text{3H}}_{2} {\text{O}}_{{({\text{aq}})}}  + {\text{AgNO}}_{{3({\text{aq}})}}  + \left( {{\text{C}}_{6} {\text{H}}_{{10}} {\text{O}}_{5} } \right)_{{\text{n}}} \xrightarrow{\Delta }{\text{Ag}}/{\text{CuO}}_{{({\text{s}})}}  + {\text{2 NO}}_{2}  + {\text{nCO}}_{2}  + {\text{nH}}_{2} {\text{O}} + {\text{NH}}_{3}    $$

### Characterization of Ag/CuO nanoparticles

The chemical compositions of CuO, as well as Ag/CuO NPs, were authenticated utilizing an FT-IR device (Thermo Electron Corporation, model: Nicolet 6700, Waltham, MA) by a uniform spectrum resolution of 6 cm^−1^ across the transmittance and absorbance mode spectrum of 4000–400 cm^−1^. The reflection-mode XRD patterns of Ag/CuO NPs were captured at a scanning rate of 0.04 degree/s using a Shimadzu LabX-XRD-6000 with CuKα emission (λ = 1.54 Å). XRD Tube operated at 30 mA and 30 kV in the 2θ range (5°- 60°). The XRD peaks were analyzed and indexed employing a software program (*X'pert High Score*).

The surface structure and nanoscale dimensions were investigated and acquired using an SEM (scanning electron microscopy) (JEOL JSM-6360LA) instrument from Tokyo, Japan, operated at 15 kV. Furthermore, the elemental composition was determined using the EDX unit.

The optical scattered reflectance measurements were established using UV–Vis. Spectrophotometer model (Shimadzu UV-3600) in the wavelength (λ) extent (200–800 nm). The reference material was the combining sphere attachment with BaSO_4_. The proposed material was attached to the specially designed holder, and Ag/CuO NPs were compacted inside the other holder. The holder had the same thickness as the Ag/CuO NPs.

JASCO UV–Vis double beam spectrophotometer was utilized to pursue the photodegradation process under ambient conditions.

### Photocatalytic initiating study

The pure CuO and Ag/CuO NPs activities as catalysts were investigated using RhB and Carmine dye solutions underneath UVA bright exposure. The preliminary concentrations of Carmine and RhB dyes were 100 mg/L and 10 mg/L; respectively. A 50 ml dye was combined with 10 mg of the nano-catalysts CuO and Ag/CuO at pH = 7, then mixed in a dark environment for 30 min to get adsorption/desorption equilibrium. The photoreactor system was constructed at the ECCM Company in Egypt according to the blueprints drawn out by Prof. Dr. Ibrahim S. Yahia, the company's founder and chief executive officer, and it features several safety features. A unique wooden box with dimensions of 80 cm in length, 70 cm in width, and 80 cm in height encases an ECCM photoreactor that uses UVA-visible lamps. The inside is made up of fourteen carefully managed lights, seven of which are ultraviolet-A (UVA) lamps with a wavelength of 254 nm, a length of 45 cm, and a power of 18 watts each (Made by the Philips Company), and seven of which are blue-white (BW) lamps with a wavelength greater than 420 nm, a length of 60 cm, and a power of 18 watts each (Made by the Philips Company). The merged sample was placed in a photoreactor and exposed to UVA light afterward. Samples (3 ml) were collected at 10-min intervals for 50 min during the exposure. Subsequently, the collected samples were centrifugated for 10 min at 5000 rpm to eliminate the catalyst. The prepared solutions were subsequently filtered and analyzed using UV–Vis spectrometry. The proportion of dye breakdown is determined with the provided expression [41].

1 $$ Degradation\;efficiency = \frac{Ao - A}{{Ao}} \times 100. $$

*A*_*o*_ is a primary dye absorbance, and *A* is an optical absorbance at the desired time. All absorbance values were obtained by the maximum absorption at 554 nm for RhB and 520 nm for carmine dye in the absorption spectrum.

### Antagonistic bioassay

The antibacterial characteristics of Ag/CuO NPs were examined using a range of multi-drug resistant human pathogens involving Gram-positive bacteria like *Staphylococcus aureus* (V) & *Bacillus cereus* (VI). Moreover, *Gram*-negative bacteria, for instance, *Klebsiella pneumoniae* (I), *Escherichia coli* (II), *klebsiella rhinoscleromatis* (III), and *Salmonella organisms* (IV), were screened. Additionally, fungal cells like *Candida glabrata* (VII) and *Candida albicans* (VIII) were also utilized for testing. The Bioprocess Development Department, SRTA-City Alexandria, Egypt, kindly provided these human pathogens.

In this experiment, an antimicrobial efficiency of Ag/CuO NPs at numerous dosages (20, 40, 60, 80, 100, and 120 µg/mL) was screened against some human pathogens such as K.pneumoniae (I), E.coli (II), K. Rhinoscleromatis (III), Salmonella organisms (IV), Staphylococcus aureus (V), Bacillus cereus (VI), Candida glabrata (VII), and Candida albicans (VIII) using agar well-diffusion method [42]. The LB agar medium was equipped and sterilized in an autoclave for 20 min at 121 °C before being poured into Petri dishes (14 cm). Wells were sliced with a cork-borer, then filled with different Ag/CuO NPs doses and incubated at 4 °C for 4 h. The pathogens were then spread on these plates and cultured for 48 h at 37 °C. under static restrictions. Finally, the dimension of the clean zone was calculated and stated in millimeters (mm).

MIC (The minimum inhibitory concentration) of Ag/CuO NPs was then determined using the dilution broth method. The minimum microbicide concentration, the lowest dose capable of killing 99.99% of the initial microbial inoculums, was also calculated for the tested bacterial and fungal cells coded MBC and MFC, respectively. In brief, the microbial turbidity produced was evaluated using the LB broth medium. In brief, 50, 100, 150, 200, 250, 300, and 350 μg/ml of Ag/CuO NPs were mixed with 2X10^5^ CFU/mL of pathogens within 50 mL of LB-broth medium for 48 h at 30 °C for fungal cells as well as 37 °C for bacterial cells under 200 rpm. These microbial Ag/CuO NPs cultures' optical density (OD) was periodically measured at 600 nm [43, 44]. Using the formula (Eq. 2), the relative inhibitory potency (%) for microbial growth was calculated by averaging the control and treatment readings (three replicates for each) for all pathogens [45]. These trials were conducted thrice, and the outcomes were displayed as the average and standard deviation (mean ± SD).

2 $$ Relative\;inhibitory\;potency \left( \% \right) = \left( {\frac{Control - Treated}{{Control}}} \right) \times 100. $$

## Results and discussion

### FTIR analysis

**Fig. 1:**
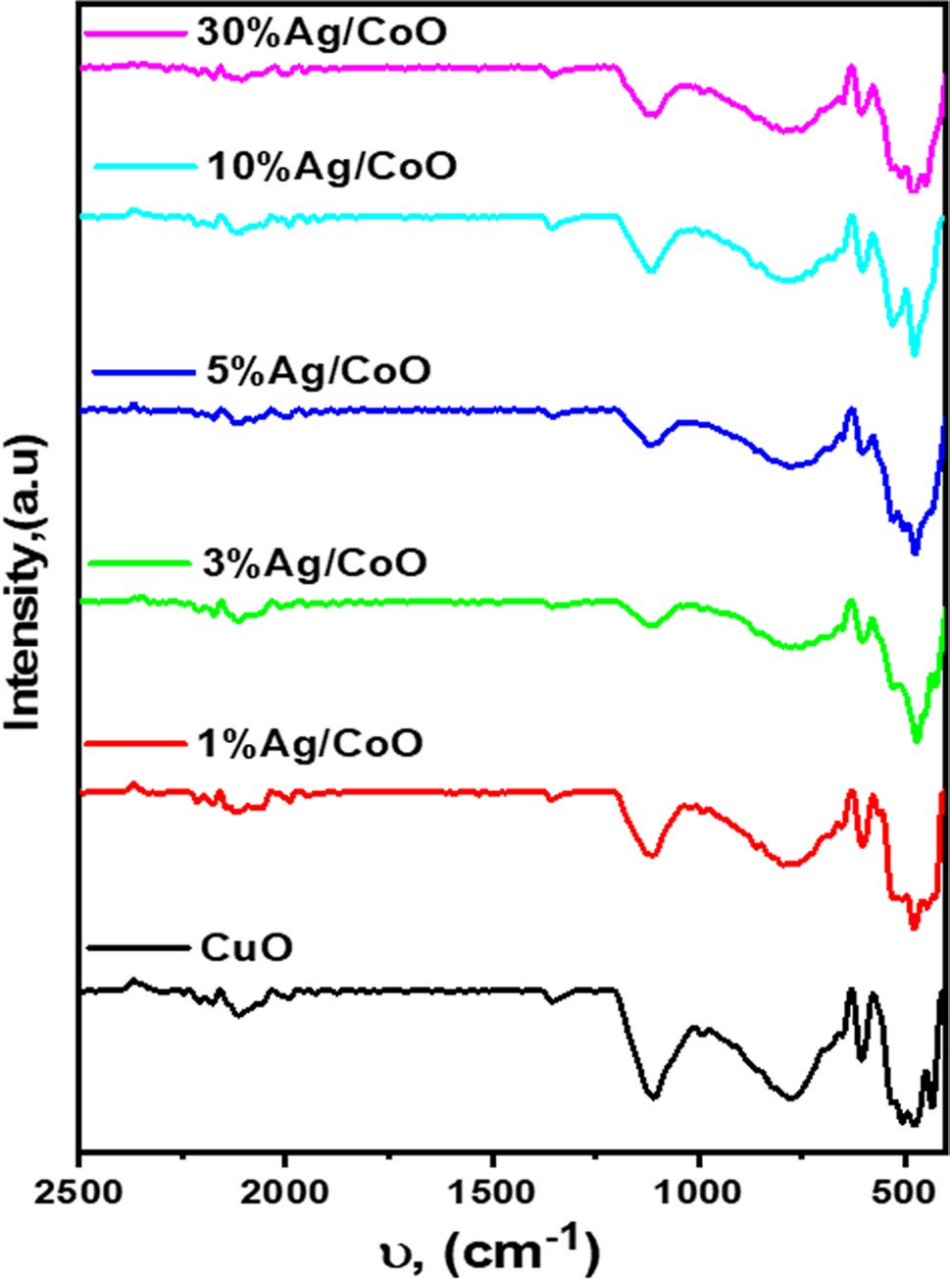
Spectral investigation by FTIR of pure CuO and different Ag/CuO NPs ratios

FTIR analysis provides the spectra of CuO's purity, surface alteration, and various proportions of Ag/CuO nanoparticles. Figure 1 shows the FTIR spectrum of Ag/CuO NPs: the broadband at ν 507, 530, and 604 cm^−1^ might be ascribed to the vibrational peak for CuO (Cu^2+^–O). These results align with existing literature, as a distinct single band for the Cu^1+^–O bond and a trio-band for the Cu^2+^–O bond were detected in ν 649, 605, and 432 cm^−1^ regions, respectively [46, 47]. When silver is added to the surface of CuO, the CuO peak at ν 995 cm^−1^ shifts to a minimal wavelength side (λ) next to ν 987 cm^−1^ [35] owing to the C–O stretching band [48]. Absorption peaks at ν 1359 cm^−1^ are haunted by the extending vibrational peak of C=C [49].

### XRD analysis

**Fig. 2:**
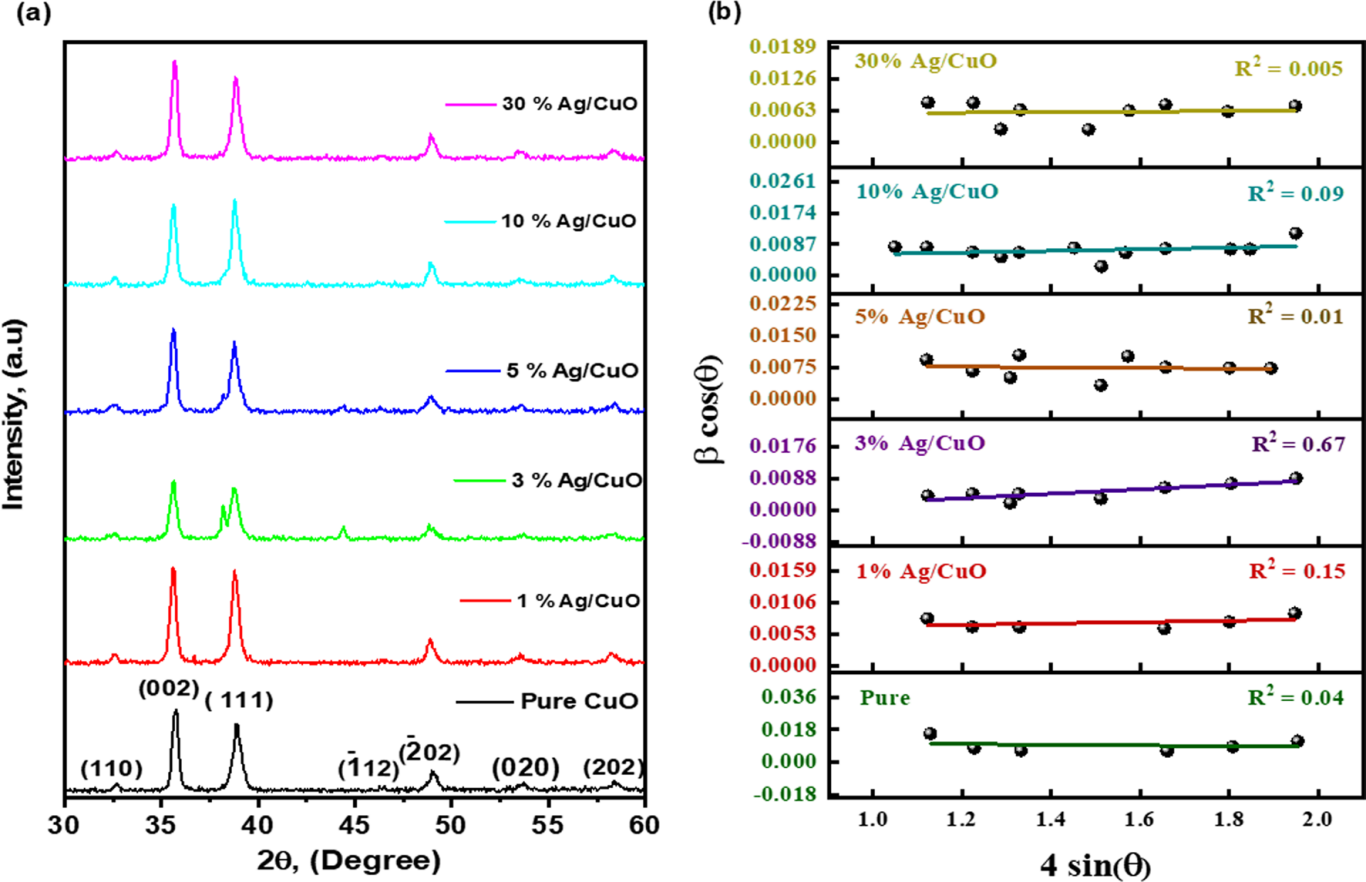
**a** XRD pattern of pure CuO and different ratios of Ag/CuO NPs, and **b** W–H Plot of pure CuO and Ag/CuO NPs

XRD patterns of pure CuO and Ag/CuO NPs were captured to determine the crystalline phase and level in the sample under examination. As shown in Fig. 2, all the nanostructures exhibit characteristic planes of pure CuO at *2θ* ~ 32.78°, 35.71°, 38.90°, 46.25°, 49.06°, 53.74°, and 58.48° as (110), (002), (111), ($\overline{1 }$12), ($\overline{2 }$02), (020), and (202) indexed to the monoclinic arrangement of CuO, which matched with typical JCPDS data (JCPDS card No: 03-065-2309) [50]. Furthermore, functionalization of Ag into the surface of CuO as illustrated through the spectrum pattern Fig. 2, Ag/CuO NPs band at 2θ ~ 38.18°, and 44.40°, which represent the planes (1 1 1) and (2 0 0) and the other peaks correspond to CuO NPs. The existence of Ag is poorer related to CuO, owing to their concentration and reactivity. Importantly, the intensive peak at 38° corresponding to the (1 1 1) plane indicates the presence of both Ag- and CuO NP phases [35]. The observed peaks of Ag/CuO were in excellent agreement with the regular JCPDS information (JCPDS card No.: 01-087-0720) [51]. Meanwhile, the crystallite size range for CuO and Ag/CuO NPs was determined with the *Debye–Scherrer* formula using *Eq. *(3) [52]:

3 $$ D = \frac{k \lambda }{{\beta {\text{Cos}} \theta }}. $$

To determine crystallite size, William Son-Hall's equation is used (4);

4 $$ \beta_{hkl} \cos \theta = \frac{k \lambda }{D} + 4\varepsilon \sin \theta . $$

wherever, *k*: *Scherer* constant (*k* = 0.9), *λ*: wavelength in the nm unit, *β*: width peak at half maximum,* θ: Bragg's* angle (degree), and D: crystallite size. Meanwhile, $\delta$: The average dislocation density and *ε*: micro-strain for CuO and Ag/CuO nanostructures were estimated with Eqs*. *(5 and 6) [52].Williamson-Hall plot is constructed by taking 4 sinθ along X axis and β cosθ along the Y axis, and the plot is linearly fitted and shown in Fig. 2b. The crystallite size of nanostructures was estimated through the intercept of the W–H plot.

5 $$ {\updelta } = { }\frac{1}{{D^{2} }} $$

6 $$ \varepsilon = \frac{{\beta\, {\text{Cos}} \theta }}{4} $$

The structural parameters like (*D*), (*δ*), and (*ε*) of synthesized CuO, as well as Ag/CuO NPs, are presented in Table 2. The dimensions are 17.24 for CuO and 25.24 nm for Ag/CuO NPs. The enhanced size influences the crystal growth, and the lattice orientation of the surface of CuO NPs was altered through Ag clusters or atoms [53, 54]. The ε and *δ* have been calculated and tabulated in Table 1. The increase in lattice strain (2.24E−03) at 10% Ag/CuO is owing to a dopant concentration producing a decrease in the dislocation density (7.237E−04 (nm)^−2^).

**Table 2:** The average crystal size, dislocation density, and microstrain values were calculated using XRD spectra for pure CuO and Ag/CuO NPs

Samples	D Scherer (nm)	D (W–H) (nm)	(*δ*) (W–H) (nm)^−2^	Lattice strain (W–H)
Pure CuO	17.246	10.980	8.294 E−03	− 2.10E−03
1% Ag/CuO	20.233	25.30	1.562 E−03	1.15E−03
3% Ag/CuO	25.463	32.856	9.263E−0.3	6.25E−03
5% Ag/CuO	17.110	16.3789	3.727E−03	− 7.19E−04
10% Ag/CuO	18.632	37.17	7.237E−04	2.24E−03
30% Ag/CuO	19.849	26.21	1.455E−03	5.12E−04

### SEM and EDX investigation

**Fig. 3:**
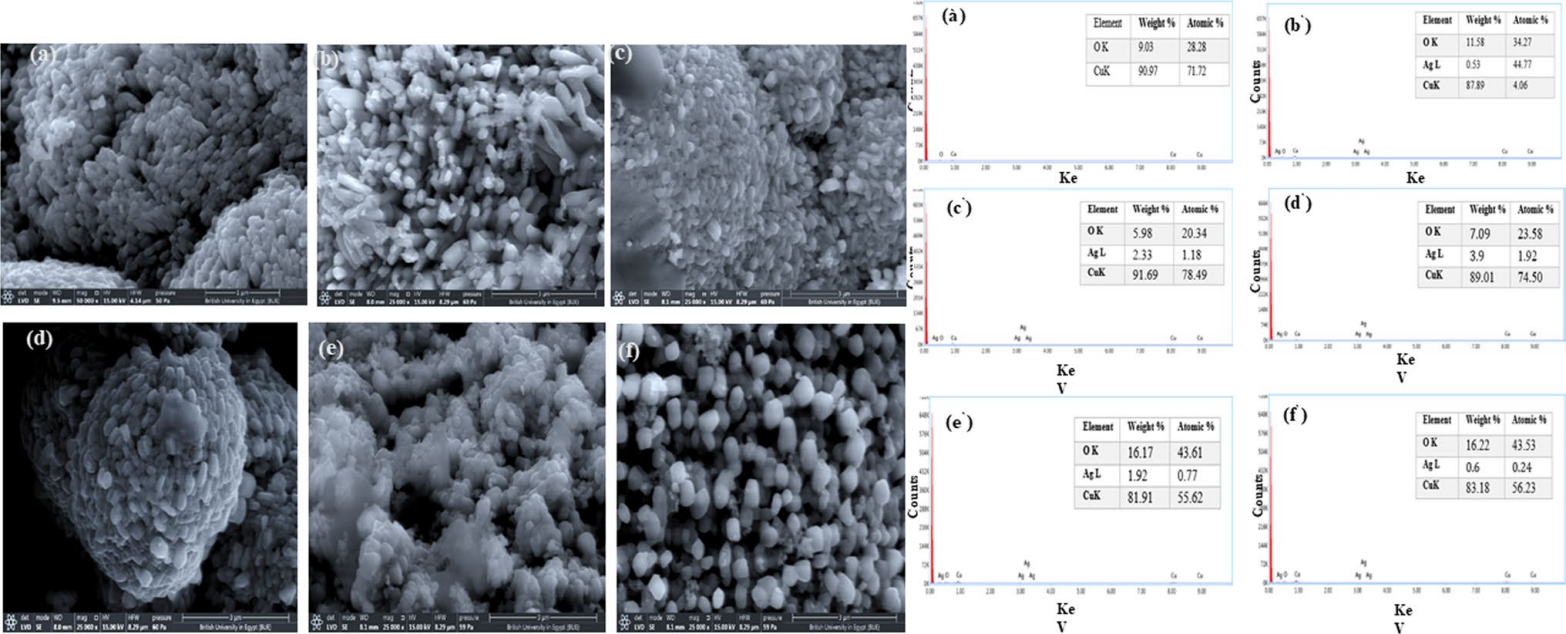
SEM images of **a** CuO in a pure state, **b**–**f** Ag/CuO NPs (original magnification 25000X, scale 3 µm, and 15 kV); and EDX analysis of **a**^**′**^ pure CuO, and **b**^**′**^–**f**^**′**^ Ag/CuO NPs

The surface construction, elemental composition, and dimensions of all fabricated CuO as well as Ag/CuO NPs were examined with EDX, as shown in Fig. 3. CuO NPs demonstrate the nano rod-shaped structure with an average particle size of ~ 100 nm along with some agglomerates Fig. 3a. The findings indicate that Ag/CuO NPs exhibit irregular spherical forms in size range of approximately 112–58 nm, along with some clusters owing to their large surface area Fig. 3b–f. The size modification in Ag/CuO NPs results from Ag^+^ (silver ions) that align with the copper oxide (Cu^2+^ and O^2−^) surface. This occurs through the lattice oxygen vacancy occupied with ions of Ag^+^ and Cu^2+^ on the surface [55]. The chemical arrangement of the different materials produced by CuO and Ag/CuO NPs was evaluated by EDX, as shown in Fig. 3a^′^–f ^′^. For basic CuO, only dual peaks of oxygen (O) and copper (Cu) are revealed to verify the formation of CuO. The doped phases (Ag/CuO) EDX spectrum proves that Ag-doped CuO with different ratios involve oxygen, copper, and silver, confirming XRD analysis results in Fig. 2 a.

### HR-TEM

**Fig. 4:**
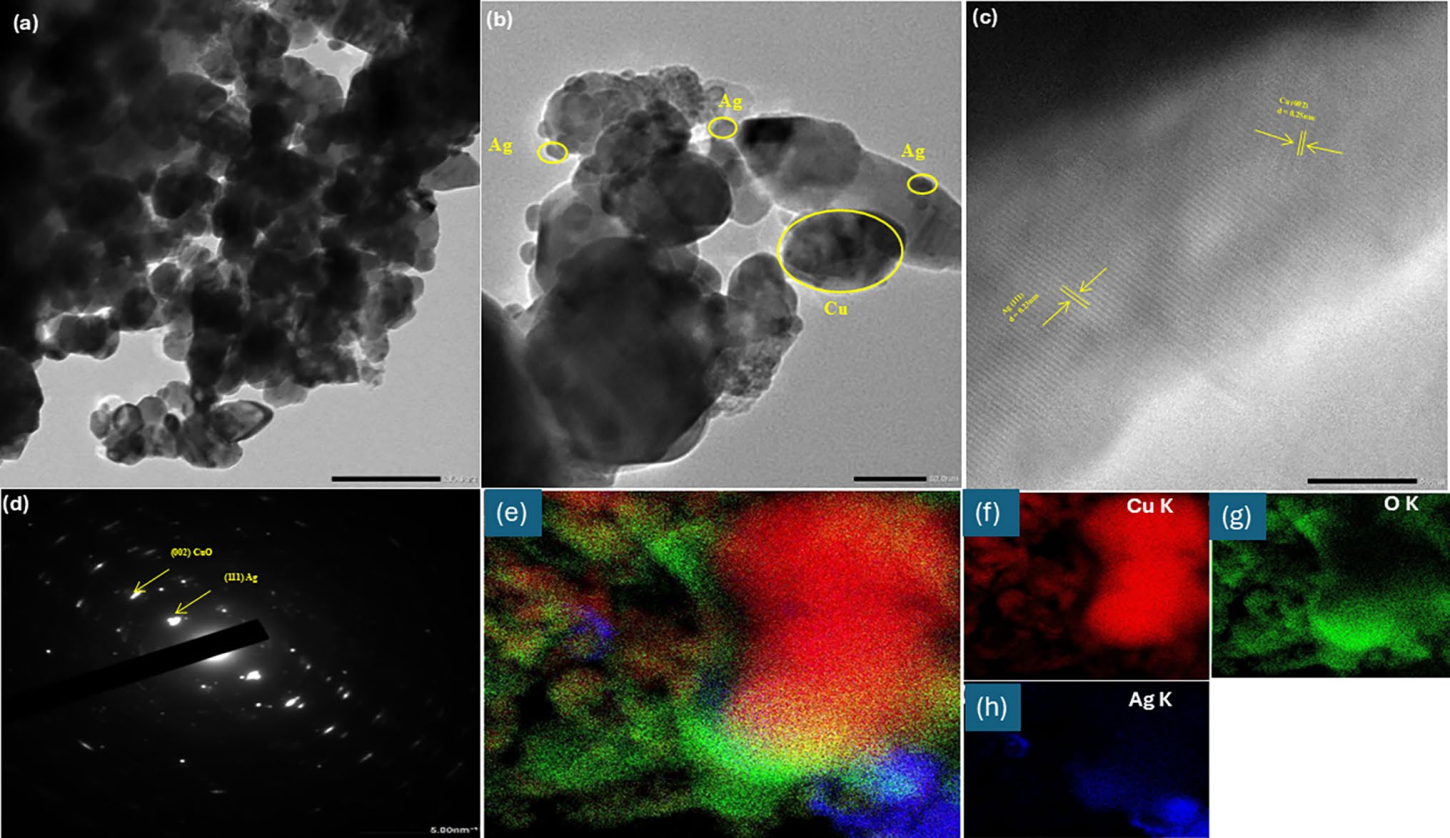
**a**, **b** TEM images of Ag/CuO NPs with different magnification, **c** HR-TEM, **d** SAED pattern, and **e**–**h** elemental mapping of Ag/CuO NPs

HRTEM analysis is used to characterize further the morphology of 10% Ag/CuO NPs (Fig. 4). The produced Ag/CuO NPs have a variety of morphologies, including spherical and oval forms, as shown in HR-TEM pictures. Ag/CuO NPs have a size of 18–25 nm, as shown in Fig. 4a, b. This study is attributed the growth of Ag/CuO NPs to starch. Ag^+^ ionic potentials exhibit greater stability than Cu^2+^ ions, and the morphology indicates the creation of Ag clusters on the CuO surface. The measurements of 0.25 nm of the (002) plane and 0.23 nm of the (111) plane for CuO and Ag; respectively, are revealed by the lattice fringes. They created Ag decorating phase on the surface of CuO, due to their atomic orientation relationship, and the values of the lattice fringes are nearly equal to those of Ag and CuO, respectively (Fig. 4c). [35]. Ag/CuO NPs' polycrystalline nature is demonstrated by the SAED pattern rings, as seen in Fig. 4d, which supports the findings of the XRD study in Fig. 2a. Using EDX mapping analysis, the atomic configuration of Ag/CuO NPs is determined. The Ag/CuO NPs are displayed in Fig. 4e–h. Copper and oxygen are richer than silver when combined. Moreover, mapping analysis Fig. 4e–h displays silver embellished with copper and oxygen atoms.

### Optical properties

**Fig. 5:**
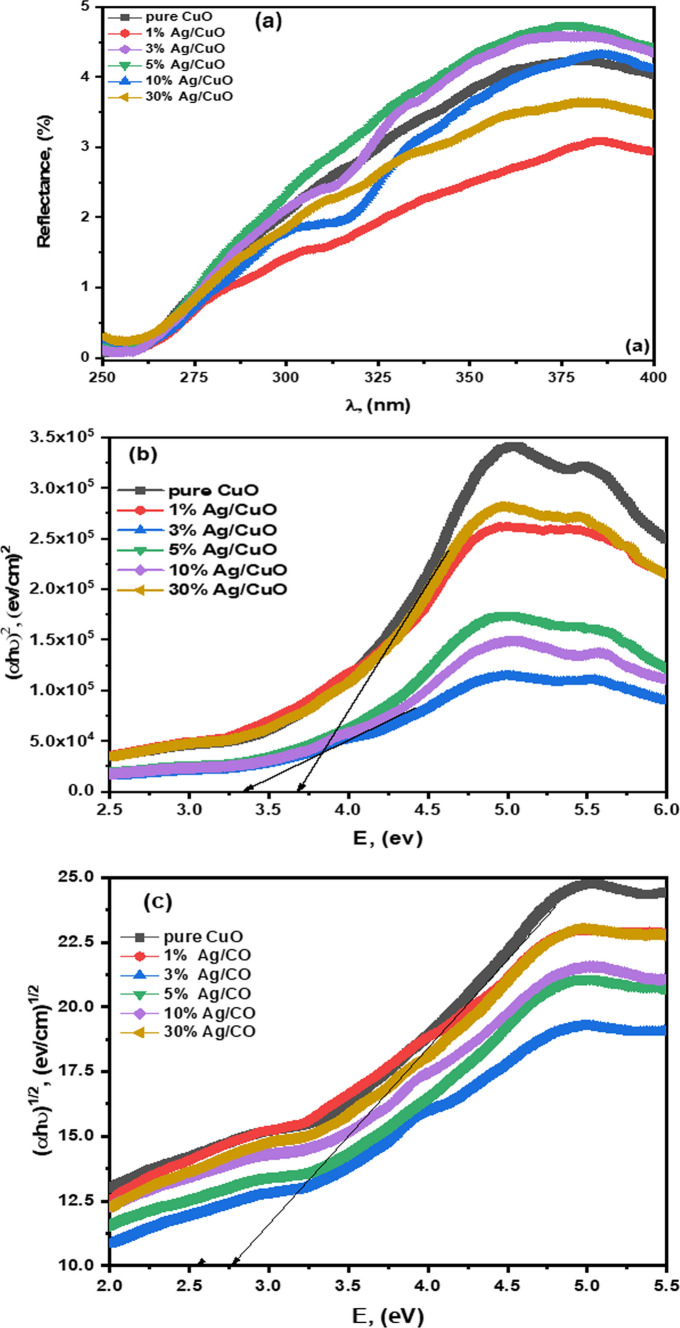
**a** UV–vis DRS spectrum range of CuO NPs and Ag/CuO NPs, **b** direct allowed bandgap, and **c** indirect allowed bandgap

The diffuse reflectance spectral (DSR) mode recorded the absorption spectrum. Figure 5a displays the absorption spectra of pure CuO and Ag/CuO NPs. The absorption edge is observed at 260 nm (i.e., UV region) for pure CuO [56]. Meanwhile, the absorbance values of Ag/CuO NPs are shifting towards the visible region because of the surface plasmon resonance (SPR) impacts of Ag/CuO NPs, which appear in Fig. 5a. The SPR was made owing to free electrons in Ag/CuO NPs. Also, in Ag/CuO NPs, the free electron of Ag/CuO NPs is done through the resonant oscillation of conduction bands interacting with specific wavelengths [57]. The optical absorption range of the NPs is dominated by the SPR effect and band shift position based on the size, structure, and aggregation state of the NPs [58]. To calculate the optical energy gap of pure CuO NPs, Ag/CuO NPs utilizing *Tauc's* equation [59]:

7 $$ \left( {\alpha h\nu } \right)^{n} = A\left( {h\nu - E_{g} } \right). $$

where *hυ*, *α*, A, and *E*_*g*_ represent the frequency of incident light, the coefficient of absorption, a constant independent of energy, and the energy for the band gap, respectively. Additionally, the quantity of (n) varies based on the nature of the optical transition in a semiconductor and is set to 1/2 and 2 for an indirect, direct bandgap semiconductor. Figure 5b, c reveal the direct and indirect bandgap semiconductors. The energy band gaps are listed in Table 3. The UV absorption of Ag/CuO NPs slightly red-shifted compared to pure CuO due to the quantum confinement effect. The reduction in energy gap enhances the electron's motion towards the holes arranged on the CuO surface, potentially increasing the oxidation process. The SPR leads to the scattering potential, the proficiency to penetrate radiation, and provides reduction profiles to the surface [35].

**Table 3:** The direct and indirect bandgap of pure CuO, Ag/CuO NPs, and comparative results with recent literatures

Samples	E.g., (direct), (eV)	E.g., (indirect), (eV)	References
Pure CuO	3.68	2.77	Present work
1% of Ag/CuO	3.63	2.61	
3% of Ag/CuO	3.50	2.65	
5% of Ag/CuO	3.39	2.72	
10% of Ag/CuO	3.34	2.55	
30% of Ag/CuO	3.61	2.64	
Ag/CuO	–	2.06	[60]
Ag/CuO	3.88	–	[61]
Ag/CuO	2.82	–	[62]
Ag/CuO	3.12	–	[63]

### Photocatalytic study of Ag/CuO

**Fig. 6:**
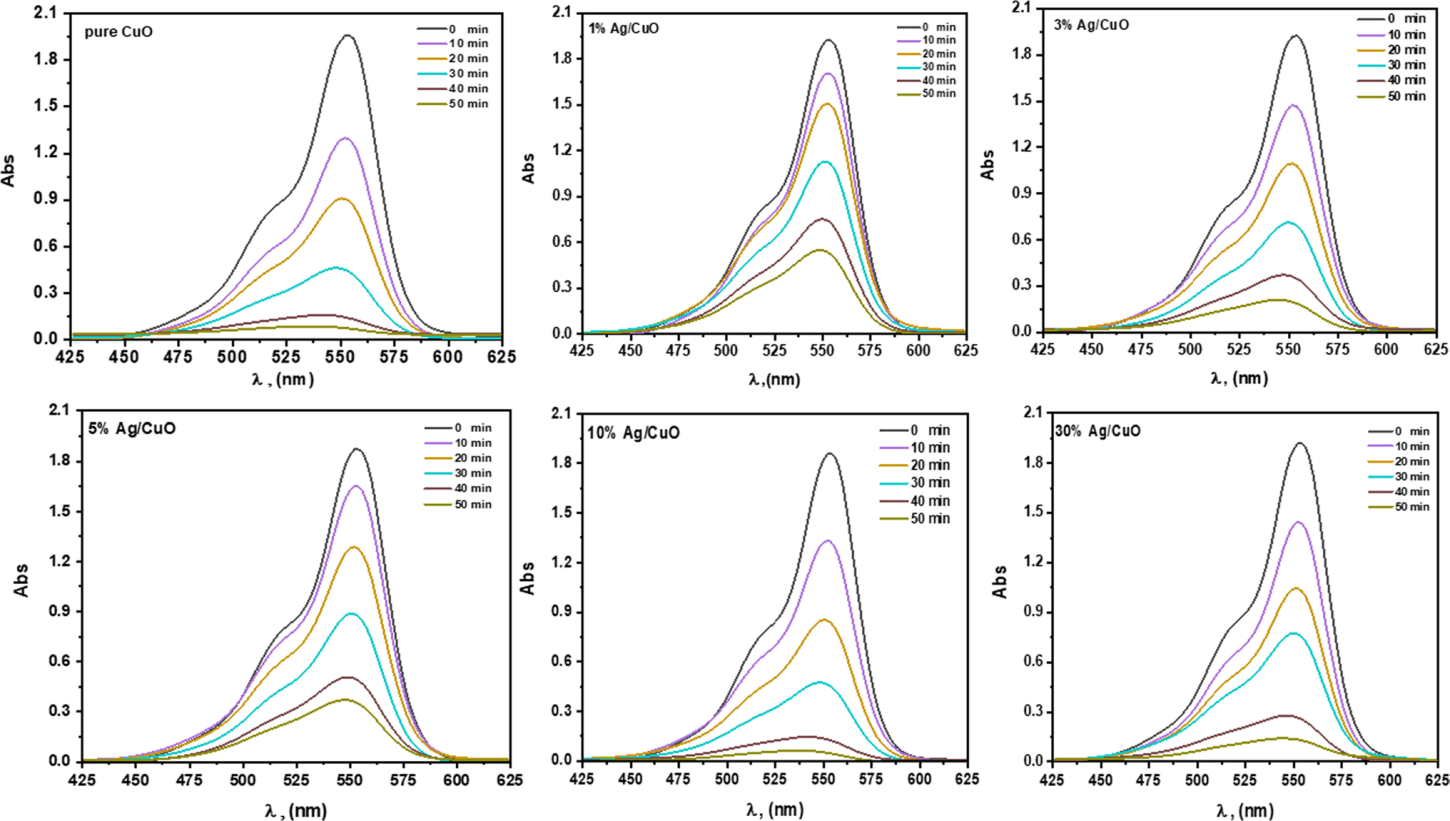
UV–Vis spectrum of RhB in the occurrence of CuO, Ag/CuO NPs at various exposure durations

**Fig. 7:**
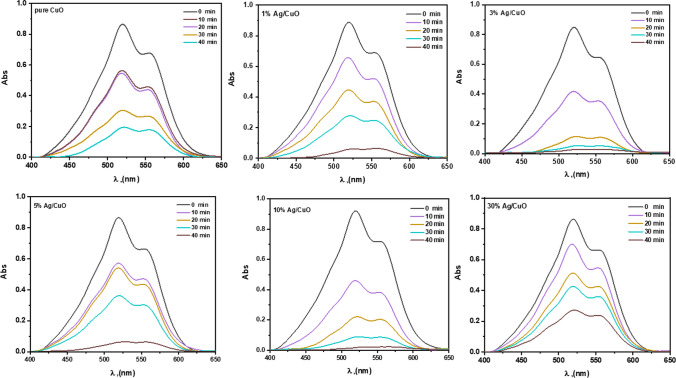
UV–visible spectrum of *Carmine* dye in the presence of CuO and Ag/CuONPs in different irradiation times

The activity of degrading photocatalytic dyes such as *Rhodamine B (RhB)* and Carmine using hybrid Ag/CuO NPs was demonstrated under UVA-light irradiation. Under UVA light, the photocatalytic mechanism of Ag/CuO NPs can generate pairs of electron–hole in the valence band (VB), as well as the conduction band (CB) of the dyes involved in the oxidation process, ultimately leading to the breakdown of the Azo dyes [64]. As shown in Figs. 6 and 7, the observable maximum absorbance of RhB at λ_max_ 554 nm and carmine dye at λ_max_ 520 nm. When irradiation time increases, the maximum absorbance decreases gradually. After 50 min, *RhB* degradation reached 93.7% for pure CuO, while 10% Ag/CuO NPs degraded *RhB*, which ranged to 98.9% simultaneously. Carmine dye degradation is detected at 77.4 and 97.8% of pure CuO and 10% Ag/CuO NPs, respectively. Also, the kinetics parameters for the dye degradation are discussed well.

**Fig. 8:**
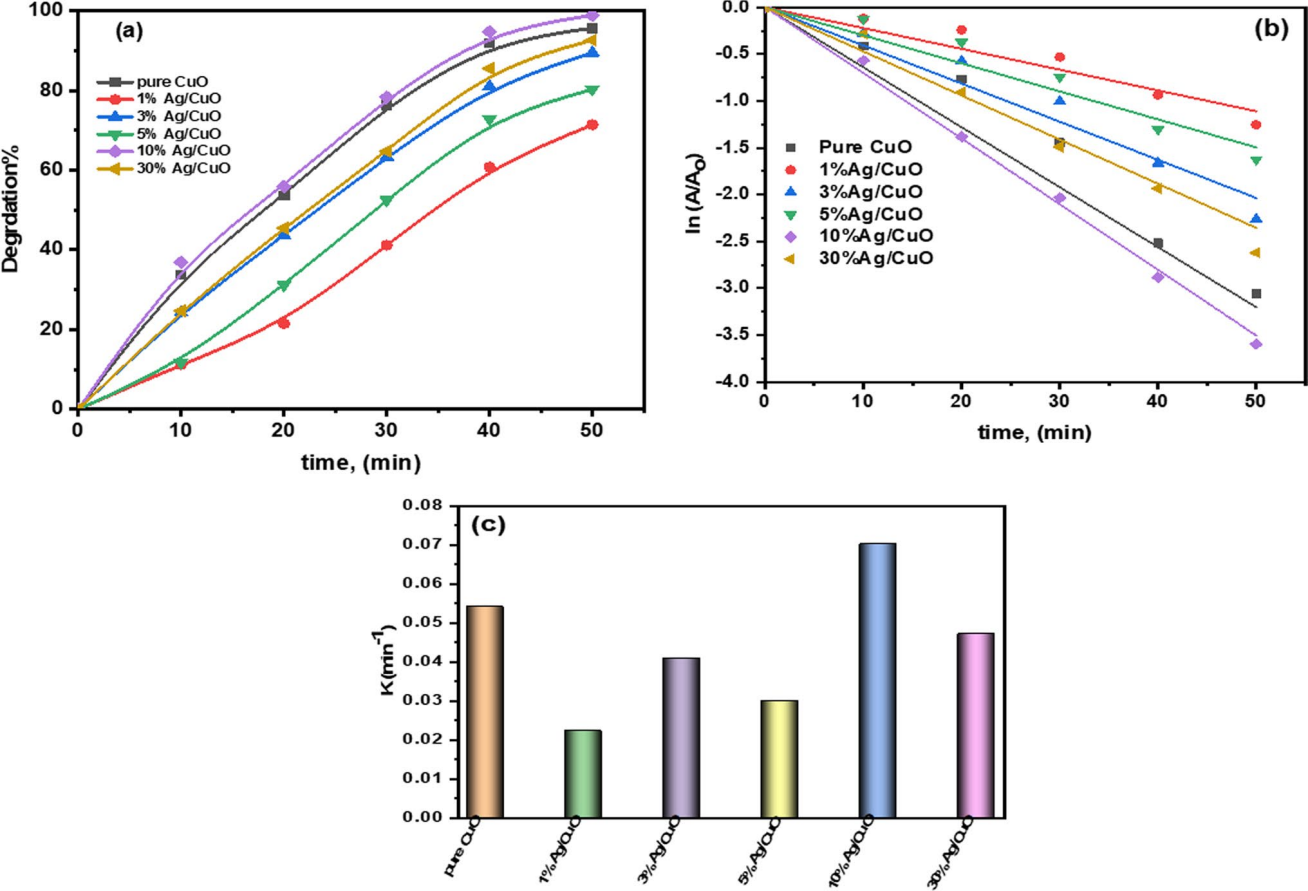
**a** Degradation efficacy of RhB, **b** kinetic rate of degradation, and **c** constant rate of reaction

**Fig. 9:**
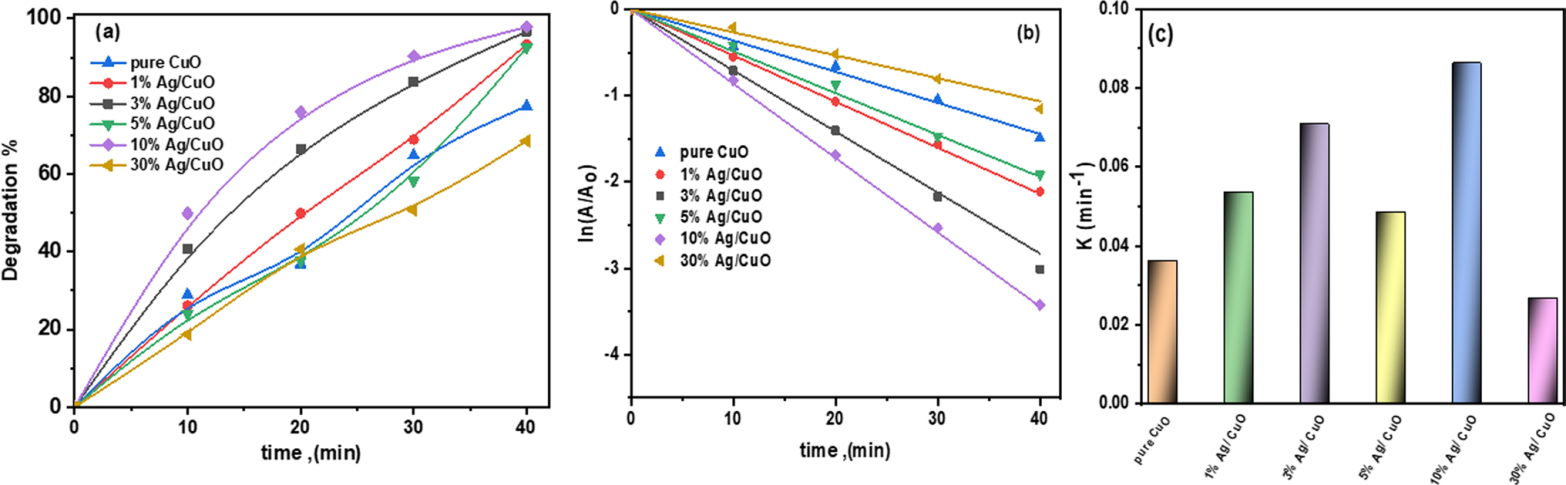
**a** Degradation efficacy of Carmine dye, **b** kinetic rate of degradation, and **c** constant rate of reaction

The photodegradation reaction by Ag/CuO NPs fits approximately with the liner given equation [65, 66]:

8 $$ {\text{ln}}\left( \frac{A}{Ao} \right) = - kt. $$

where *A*_*o*_ is a primary dye absorbance, *A* is an absorbance at the desired period, t is time, and k is a constant rate. The degradation % of RhB and Carmine dyes throgth prepared composites are presented in Figs. 8a and 9a; respectively. The quantities of *k*, which are the slopes of the lines achieved, are calculated and are found at 0.0540 and 0.0701 min^−1^ for pure CuO, while 10% of Ag/CuO NPs under RhB dye are shown through Figs. 8b, c and 9b, c, the kinetic rate of degradation of carmine dye is 0.0363, 0.0863 min^−1^ for pure CuO, and 10% of Ag/CuO NPs, as summarized in Table 4. The proposed *R*^*2*^ value is found to be more than 0.9, saying that as a function of time, the photo breakdown reaction couples the pseudo-first-order kinetics. In contrast, the *k* value can be obtained from the given calculation. The optimum degradation efficiency and high kinetics rate are noted for the 10% of Ag/CuO NPs sample. Since 10% of Ag/CuO NPs have a low bandgap, they can absorb light at their greatest rate. This may account for the smaller particle size, electron/hole recombination, and high activity. According to UVA research, the bandgap increases for 30% Ag/CuO NPs, which lowers the nanomaterial’s absorption and, eventually, its degradation potential. As shown in Table 5, 10% of the Ag/CuO NPs are compared with the previous results in literatures for photodegradation of dyes.

**Table 4:** Photo-degradation efficiency and rate constant and *R*^2^ value of different samples for RhB dye

Samples		Pure CuO	1% Ag/CuO	3% Ag/CuO	5% Ag/CuO	10% Ag/CuO	30% Ag/CuO
Degradation efficiency	RhB	93.7%	71.4%	89.5%	80.3%	98.9%	92.7%
	Carmine	77.4%	92.5%	96.6%	93.3%	97.8%	68.4%
K (min^−1^)	RhB	0.0540	0.0221	0.0407	0.0299	0.0701	0.0471
	Carmine	0.0363	0.0534	0.0709	0.0483	0.0863	0.0267
R^2^	RhB	0.98	0.96	0.98	0.97	0.99	0.99
	Carmine	0.99	0.99	0.97	0.99	0.99	0.99

**Table 5:** A comparative study with literature on Ag/CuO NPs' performance for photodegradation was conducted

Catalyst	Pollutant, Conc	Irradiation light	Degradation (%)	Time	References
Ag/CuO	[RhB] = 30 mg/L	Xenon lamp	92	120 min	[35]
Ag/CuO	[MB] = 15 mg/L	tungsten lamp, 300 W	98.8	4 h	[67]
Ag/CuO	[MB] = 40 mg/L	Xenon lamp	86	40 min	[62]
Ag/CuO	[MB] = 0.05 mg/L	UV light	98	120 min	[68]
Ag/CuO	[AO] = 0.01 mg/L	–	92.77	60 min	[69]
10% Ag/CuO	[RhB] = 30 mg/L	UVA light	98.9	50 min	Present work
	[Carmine] = 20ppm		97.8	40 min	

### Photocatalytic degradation mechanism of RhB dye

**Fig. 10:**
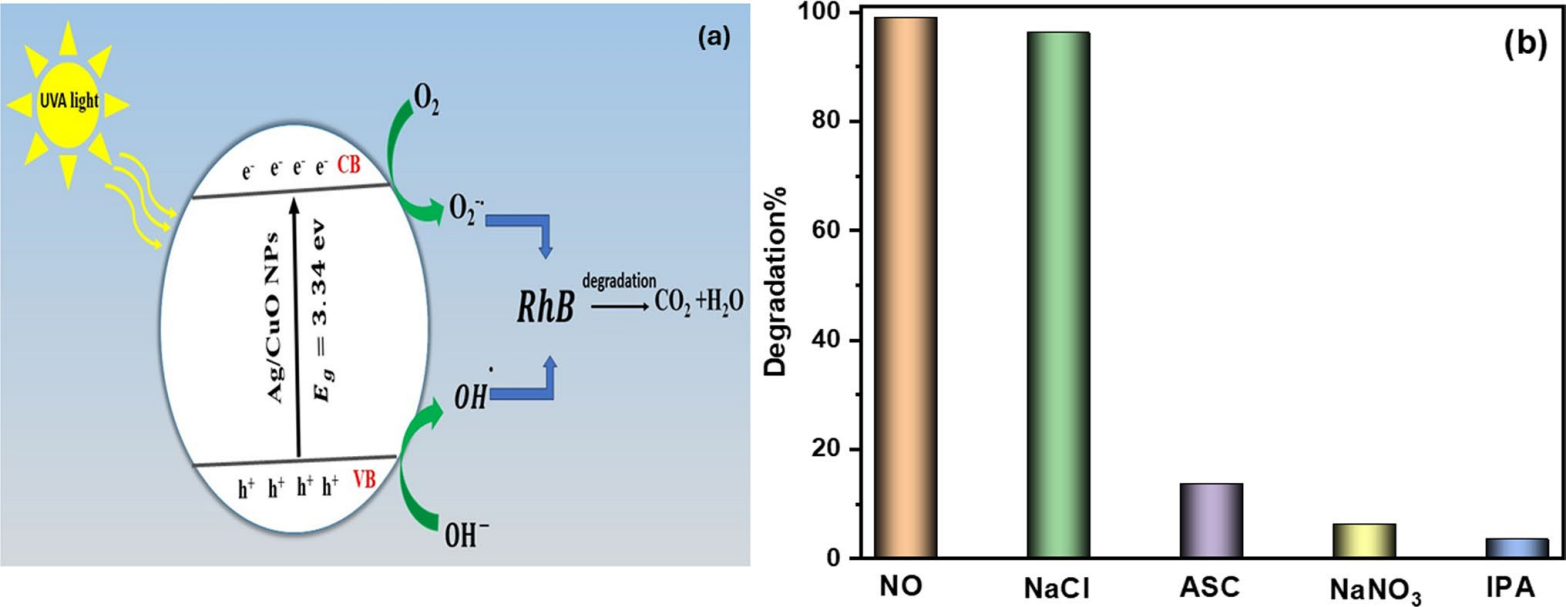
**a** The mechanism of RhB photodegradation over Ag/CuO NPs underneath UVA-light radiation, and **b** The impact of scavengers on the photocatalytic breakdown of RhB underneath UVA light

Trapping experiments for reactive species were conducted to gain a thorough understanding of the fundamental photocatalytic mechanism. It was observed that superoxide radicals (O_2_^·−^), holes (h^+^), and hydroxyl radicals (·OH) could be produced during the degradation of organic dyes [70]. The isopropanol (IPA), sodium nitrate (NaNO_3_), sodium chloride (NaCl), and ascorbic acid (ASC) served as hunters for the radicals ^·^OH, e^−^, H^+^, and O_2_^·−^, respectively [71]. The degradation rate noticeably decreased upon the addition of specific trapping scavengers. This decrease in the degradation of RhB is attributed to the absence of involvement in the dye degradation by the specific trapping scavengers, as shown in Fig. 10a.

Consequently, different trapping scavengers can be employed to identify the primary radicals that participate in photocatalytic reactions. In Fig. 10b, the addition of IPA to the reaction solution followed by a decrease in the photocatalytic degradation degree of the RhB solution from 98.9 to 5.5%, confirming that hydroxide radicals play a major role in the photocatalysis process. Furthermore, the introduction of NaNO_3_ had an inhibitory effect on the RhB degradation rate, which still reached 6.5%, indicating a limited contribution of electrons to the removal of RhB. The addition of ASC led to a decrease in degradation to 14%. The results of the radical scavenging experiment demonstrate that (^·^OH) is a more active species and may directly oxidize the RhB dye.

After exposing the photocatalysts to sufficient light, as illustrated in Fig. 10a, the photocatalytic dye degradation process involves light absorption, the creation of electron–hole pairs, and redox reactions with the dye adsorbed on the surface of the photocatalyst. During this process, charge species such as OH^−^, h^+^, and e^−^act as oxidizing and reducing agents, and ^·^OH and O_2_^·^ radicals and holes (h^+^) are intermediates that spontaneously react with the nearby ions, degrading the dye compounds to environmentally friendly CO_2_ and H_2_O as indicated by the following Eqs. (9–13):

9 $$ {\text{Ag}}/{\text{CuO}} +  {\text{h}{\upnu}}   \to {\text{Ag}}/{\text{CuO}}\left( {{\text{h}}^{ + } } \right) + {\text{Ag}}/{\text{CuO}}\left( {{\text{e}}^{ - } } \right) $$

10 $$ {\text{h}}^{ + } + {\text{H}}_{2} {\text{O}} \to {\text{OH}}^{ - } + {\text{H}}^{ + } $$

11 $$ {\text{e}}^{ - } + {\text{O}}_{2} \to {\text{O}}_{2}^{ \cdot - } $$

12 $$ {\text{OH}}^{ - } + {\text{h}}^{ + } \to {\text{OH}}^{ \cdot } $$

13 $$ {\text{OH}}^{ \cdot } + {\text{RhB}}\;{\text{dye}} \to {\text{CO}}_{2} + {\text{H}}_{2} {\text{O}} $$

### Recycling and reusability of Ag/CuO NPs with RhB dye

**Fig. 11:**
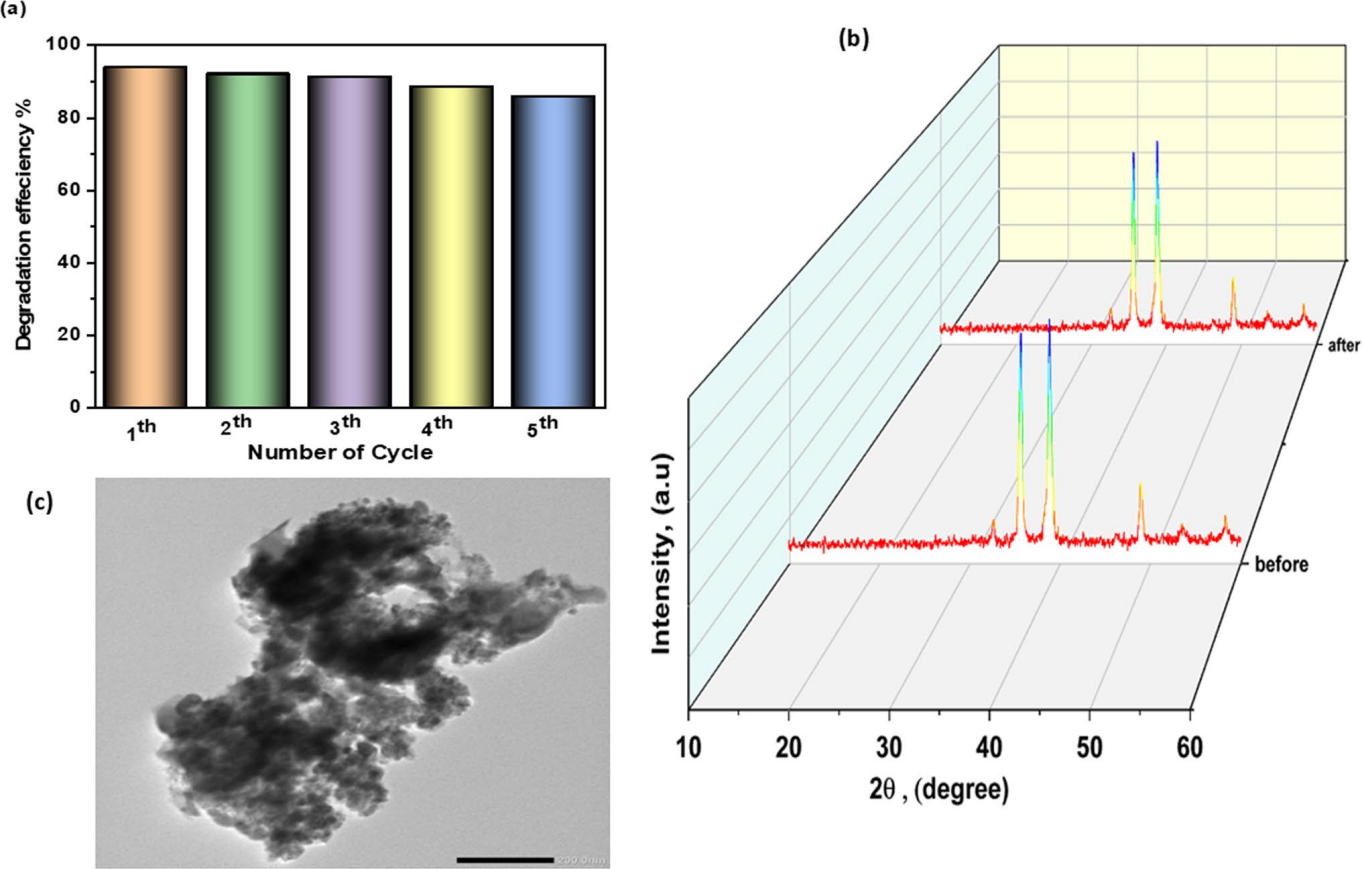
**a** The cycle experiment of 10%Ag/CuO NPs for degradation of RhB dye, **b** XRD before and after reusability, and **c** TEM after the used catalyst

To investigate the stability and reusability of nanocatalysts, it was tested by recovering photocatalysts for five cycles, as shown in Fig. 11a. The results show that the photocatalytic degradation efficiency of RhB over 10%Ag/CuO NPs was slightly weakened, reaching about 98.7% after the five cycles, highlighting the excellent stability and reusability of Ag/CuO NPs for dye degradation. XRD patterns (Fig. 11b) of the initial and reused Ag/CuO NPs indicate no alteration in the sample’s crystallinity properties. TEM images (Fig. 11c) indicate a considerable change in the recycled catalyst's morphological properties. Because of the pore blockage caused by the agglomeration of catalyst particles, the particle appears to be less rough, and the amount of agglomeration seems to be reduced. Overall, our results prove that the Ag/CuO NPs catalyst has good physicochemical stability and is reusable.

### Antimicrobial activities of Ag/CuO

**Fig. 12:**
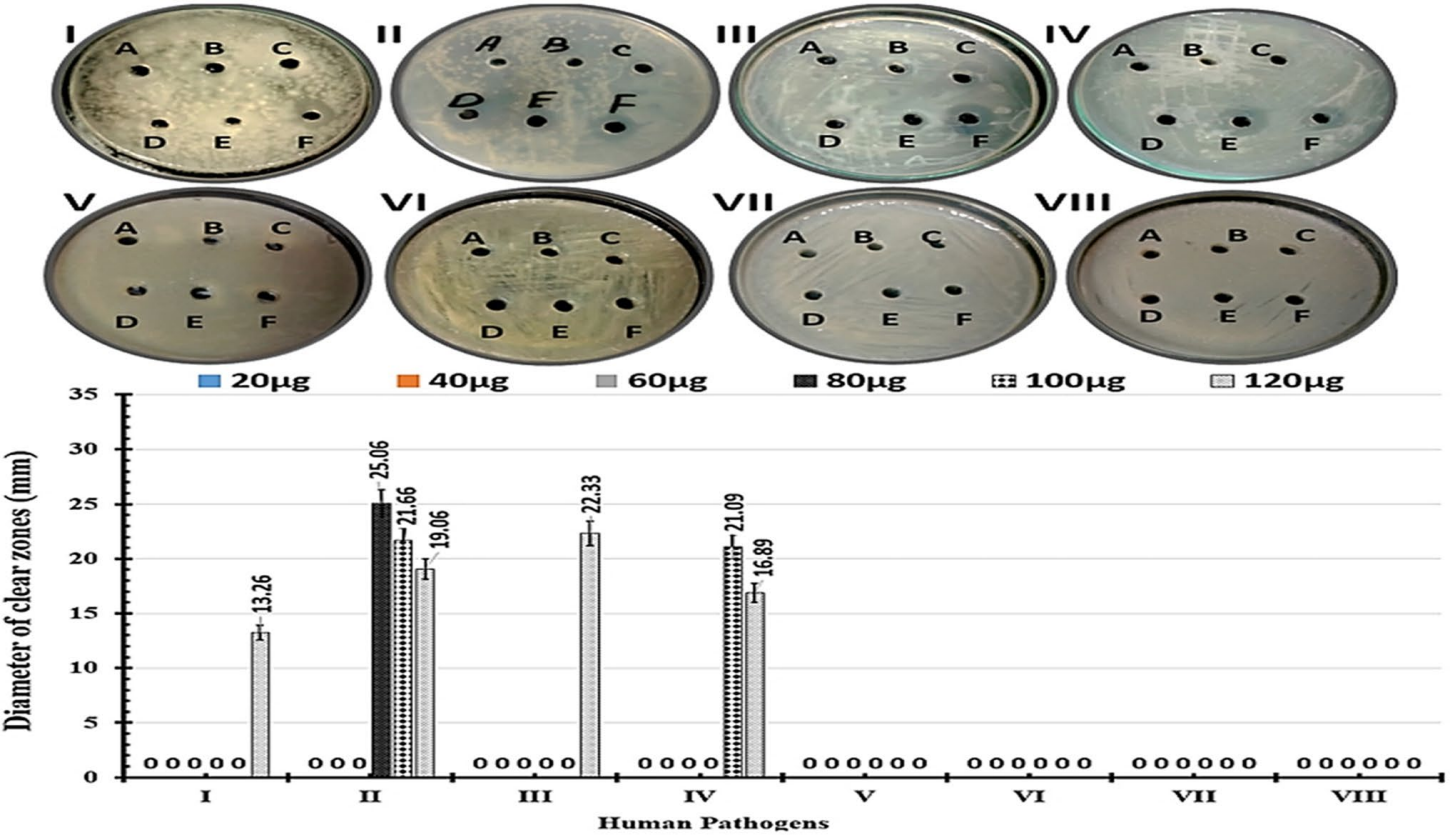
Antimicrobial bioassay of the Ag/CuONPs were carried out at different doses of (20, 40, 60, 80, 100, and 120 µg/ml) represented as (A, B, C, D, E, and F, respectively) against some human pathogens like; *K. pneumoniae* (**I**), *E.coli* (**II**), *k. rhinoscleromatis* (**III**), *Salmonella organisms* (**IV**), *Staphylococcus aureus* (**V**), *Bacillus cereus* (**VI**), *Candida glabrata* (**VII**), and *Candida albicans* **(VIII**) by calculating the Diameter of clear zones (mm)

*In-vitro* evaluation of the antimicrobial properties of different Ag/CuO NPs doses. According to Wang et al., composite nanoparticles made from multiple metal oxides can be used as more potent antimicrobial agents than single nanoparticles [72]. In this scenario, microbes find it extremely difficult to resist nano-composites since the chances of a cell having multiple resistance mutations are extremely low [72]. In our experiments, the antimicrobial efficacy of Ag/CuO NPs at several dosages (20, 40, 60, 80, 100, and 120 µg/mL) alongside some human pathogens was initially investigated utilizing the agar well diffusion method (Fig. 12). Subsequently, by the broth dilution method (Table 6 and Fig. 12). Firstly, no identifiable clear zones were identified against all tested pathogens at dosages of 20, 40, and 60 µg/mL of Ag/CuO NPs **(**Fig. 12). It is also noteworthy that all Gram-positive bacteria, as well as fungal cells, were significantly more resistive to all Ag/CuO NPs dosages than gram-negative bacteria. Table 6 shows that *E. coli* had the largest clear zone diameter (25.06 ± 0.26), followed by *K. rhinoscleromatis* (22.33 ± 1.09) and *Salmonella organisms* (21.09 ± 1.54) at 80, 120, and 100 µg/mL of Ag/CuO NPs, respectively. Meanwhile, the lowest clear zone was observed in the case of *Klebsiella pneumoniae* (13.26 ± 0.78) using Ag/CuO NPs with a concentration of 120 µg/mL **(**Fig. 12).

**Table 6:** The Diameter of clear zones showing the antimicrobial efficiency of Ag-doped-CuO NPs at various dosages against various human pathogens

Human Pathogens	Diameter of clear zones (mm ± SD)					
	Ag-doped-CuO NPs (µg/mL)					
	20	40	60	80	100	120
*K. pneumoniae*	00 ± 00	00 ± 00	00 ± 00	00 ± 00	00 ± 00	13.26 ± 0.78
*E. coli*	00 ± 00	00 ± 00	00 ± 00	25.06 ± 0.26	21.66 ± 1.45	19.06 ± 0.89
*K. rhinoscleromatis*	00 ± 00	00 ± 00	00 ± 00	00 ± 00	00 ± 00	22.33 ± 1.09
*Salmonella organisms*	00 ± 00	00 ± 00	00 ± 00	00 ± 00	21.09 ± 1.54	16.89 ± 1.07
*Staphylococcus aureus*	00 ± 00	00 ± 00	00 ± 00	00 ± 00	00 ± 00	00 ± 00
*Bacillus cereus*	00 ± 00	00 ± 00	00 ± 00	00 ± 00	00 ± 00	00 ± 00
*Candida glabrata*	00 ± 00	00 ± 00	00 ± 00	00 ± 00	00 ± 00	00 ± 00
*Candida albicans*	00 ± 00	00 ± 00	00 ± 00	00 ± 00	00 ± 00	00 ± 00

**Fig. 13:**
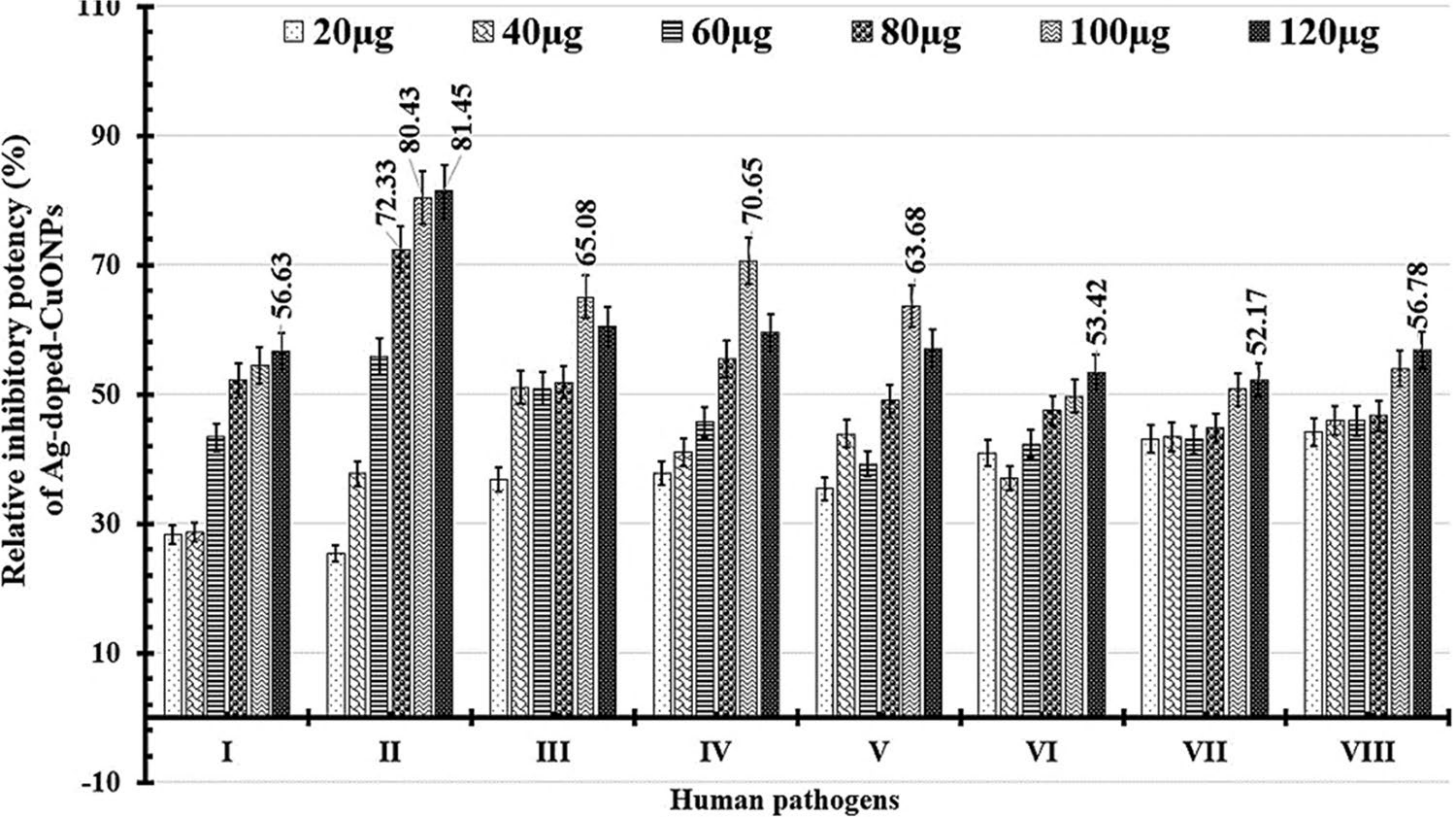
Relative inhibitory potency (%) as a parameter of the manufactured cell density (OD600nm) after treatment with different doses of Ag-doped-CuONPs for some pathogenic growth such as *K. pneumoniae* (**I**), *E.coli* (**II**), *k.rhinoscleromatis* (**III**), *Salmonella organisms* (**IV**), *Staphylococcus aureus* (**V**), *Bacillus cereus* (**VI**), *Candida glabrata* (**VII**), and *Candida albicans* (**VIII**)

Secondly, the calculated MIC of Ag/CuO NPs for all pathogens using the broth dilution method ranged from 100 to 120 µg/mL, as shown in Table 7. Noticeably, (20, 40, 60, 80, 100, and 120 µg/mL) are all the tested doses of Ag/CuO NPs which reduced all pathogenic proliferation (Fig. 13). The largest relative inhibitory activities were tabulated against *Escherichia coli* (81.45 ± 1.39%) at 120 µg/mL and at 100 µg/mL (80.43 ± 0.59%) followed by 80 µg/mL that recoded as 72.33 ± 0.82%. Additionally, the lowest relative inhibitory activities are examined against fungal cells and Gram + ve bacteria at 120 µg/mL % of Ag/CuO NPs, respectively, at 52.17 ± 1.49% and 53.42 ± 1.71%. So, *Bacillus cereus* and *Candida glabrata* need the largest MBC and MFC (300 µg/mL) of Ag/CuO NPs to kill 99.99% of the microbial cells (Table 7).

**Table 7:** Relative inhibitory potency (%) as a parameter of the produced microbial cell density (OD_600nm_) for treated pathogens with various quantities of Ag-doped-CuO NPs; additionally, the minimal inhibition (MIC), minimal bactericidal (MBC), as well as minimal fungicidal (MFC) concentrations

Human Pathogens	Relative inhibitory potency (% ± SD)						MIC (µg/ml)	MBC (µg/ml)	MFC (µg/ml)
	Ag-doped-CuO NPs (µg/ml)								
	20	40	60	80	100	120			
*K. pneumoniae*	28.32 ± 1.05	28.6 ± 1.05	43.34 ± 2.22	52.22 ± 2.91	54.50 ± 0.88	56.63 ± 0.65	120	150	
*E.coli*	25.44 ± 0.79	37.7 ± 1.39	55.82 ± 1.10	72.33 ± 0.82	80.43 ± 0.59	81.45 ± 1.39	120	200	
*K. rhinoscleromatis*	36.89 ± 1.93	51.09 ± 1.8	50.86 ± 1.34	51.85 ± 0.63	65.08 ± 1.13	60.52 ± 1.06	100	150	
*Salmonella organisms*	37.76 ± 1.87	41.00 ± 1.56	45.71 ± 1.05	55.47 ± 0.67	70.65 ± 1.54	59.46 ± 0.96	100	250	
*Staphylococcus aureus*	35.38 ± 3.4	43.89 ± 2.74	39.19 ± 1.00	48.98 ± 7.78	63.68 ± 1.11	57.12 ± 0.24	100	200	
*Bacillus cereus*	40.98 ± 1.38	37.04 ± 2.05	42.30 ± 1.05	47.42 ± 0.955	49.72 ± 0.45	53.42 ± 1.71	120	300	
*Candida glabrata*	43.08 ± 2.43	43.4 ± 2.16	43.02 ± 2.61	44.79 ± 1.02	50.72 ± 1.01	52.17 ± 1.49	120		300
*Candida albicans*	44.13 ± 3.48	45.94 ± 3.58	45.9 ± 3.46	46.69 ± 0.74	53.98 ± 2.18	56.78 ± 1.21	120		150

Despite this, the observed MBCs have a diverse variety of Ag/CuO NPs dosages, ranging from 150 to 250 g/mL, which help to kill 99.99% of all *Gram-negative* pathogenic cells evaluated, as revealed in Table 7. Sreenivasa et al*.* stated that Ag NPs can rapidly penetrate bacteria's nuclear material depending on their size, shape, and surface area, resulting in relatively high antimicrobial activity [45]. Moreover, several publications reported that metal oxide nanoparticles have antimicrobial effects by distributing metal ions that interrupt membrane permeability and attach to the surface of the microbial cell membrane [73], DNA replication, generate reactive oxygen species (ROS), and significantly contribute to bacterial cell death [72, 73]. Since the inhibitory effect could be due to nanoparticle ions interacting with DNA and RNA and subsequently interrupting replication, The inhibitory effect could be associated with a rise in the generation of ROS, which can damage proteins and enzymes involved in cellular respiration, leading to cell death [74].

## Conclusions

The outcomes of the present study revealed that the auto-combustion approach synthesizes pure CuO and Ag/CuO NPs. Shows a significant influence on the photocatalytic and antimicrobial activities of synthesized Ag/CuO NPs. Moreover, results also confirmed that the size and shape of NPs could be investigated with FTIR, XRD, SEM, and UV–VIS DRS. The FT-IR spectra of the synthesized samples confirmed the formation of Ag/CuO nanostructures. XRD patterns confirmed the high crystallinity with the monoclinic structure of all the synthesized Ag/CuO nano-samples. The chemical synthesized CuO and Ag/CuO NPs had an average crystallite size of 17.2–25.4 nm as analyzed by XRD, respectively. The SEM demonstrates the nano rod-shaped structure of CuO while adding Ag NPs, finding irregular spherical forms with an average particle size of ~ 100 nm. The optical band gap energy is pure CuO and Ag/CuO NPs. The absorption edge is observed at 260 nm for pure CuO. Meanwhile, the absorbance values of Ag/CuO NPs are shifting towards the visible region because of the impact of surface plasmon resonance (SPR) on Ag/CuO NPs.

Additionally, the photocatalytic activity of the prepared catalyst was examined through the degradation of RhB and carmine dyes under UVA-bright irradiation. Thus, Ag/CuO NPs show improved photo-induced electron–hole pair separation and better photocatalytic activity than other pure CuO NPs. The results of the radical scavenging experiment demonstrate that (·OH) is a more active species and may directly oxidize RhB dye. Besides, Ag/CuO Nps manifests superior antimicrobial activity against Gram-positive bacteria like *Staphylococcus aureus* (V) & *Bacillus cereus* (VI). Moreover, *Gram*-negative bacteria, such as *K.pneumoniae* (I), *E.coli* (II), *K.rhinoscleromatis* (III), and *Salmonella* organisms, are also present. Ag/CuO NPs can rapidly penetrate bacteria's nuclear material depending on their size, shape, and surface area, resulting in relatively high antimicrobial activity. Ag/CuO NPs are an ideal material for both the degradation of organic pollutants and the removal of bacterial strains in wastewater.

## Data Availability

The datasets used and analyzed during the current study are available from the corresponding author upon reasonable request.
